# Factors influencing agreement of breast cancer luminal molecular subtype by Ki67 labeling index between core needle biopsy and surgical resection specimens

**DOI:** 10.1007/s00428-020-02818-4

**Published:** 2020-05-07

**Authors:** Kristina A. Tendl-Schulz, Fabian Rössler, Philipp Wimmer, Ulrike M. Heber, Martina Mittlböck, Nicolas Kozakowski, Katja Pinker, Rupert Bartsch, Peter Dubsky, Florian Fitzal, Martin Filipits, Fanny Carolina Eckel, Eva-Maria Langthaler, Günther Steger, Michael Gnant, Christian F. Singer, Thomas H. Helbich, Zsuzsanna Bago-Horvath

**Affiliations:** 1 Department of Pathology and Comprehensive Cancer Center, Medical University of Vienna, 18-20 Waehringer Guertel, A-1090 Vienna, Austria; 2 Department of Surgery and Transplantation, University Hospital and University of Zurich, Zurich, Switzerland; 3 Center for Medical Statistics, Informatics, and Intelligent Systems, Section for Clinical Biometrics, Medical University of Vienna, Vienna, Austria; 4 Department of Biomedical Imaging and Image-guided Therapy, Division of Molecular and Gender Imaging, Medical University of Vienna, Vienna, Austria; 5 Department of Radiology, Breast Imaging Service, Memorial Sloan Kettering Cancer Center, New York, NY USA; 6 Department for Medicine I/Division of Oncology, Medical University of Vienna, Vienna, Austria; 7 Department of Surgery and Breast Health Center, Comprehensive Cancer Center, Medical University of Vienna, Vienna, Austria; 8 Hirslanden Klinik St. Anna Brustzentrum, Lucerne, Switzerland; 9 Institute of Cancer Research and Comprehensive Cancer Center, Medical University Vienna, Vienna, Austria; 10 Comprehensive Cancer Center, Medical University of Vienna, Vienna, Austria; 11 Department of Obstetrics and Gynaecology and Breast Health Center, Comprehensive Cancer Center, Medical University of Vienna, Vienna, Austria

**Keywords:** Breast cancer, Luminal molecular subtype, Agreement, Core needle biopsy, Ki67

## Abstract

Reliable determination of Ki67 labeling index (Ki67-LI) on core needle biopsy (CNB) is essential for determining breast cancer molecular subtype for therapy planning. However, studies on agreement between molecular subtype and Ki67-LI between CNB and surgical resection (SR) specimens are conflicting. The present study analyzed the influence of clinicopathological and sampling-associated factors on agreement. Molecular subtype was determined visually by Ki67-LI in 484 pairs of CNB and SR specimens of invasive estrogen receptor (ER)–positive, human epidermal growth factor (HER2)–negative breast cancer. Luminal B disease was defined by Ki67-LI > 20% in SR. Correlation of molecular subtype agreement with age, menopausal status, CNB method, Breast Imaging Reporting and Data System imaging category, time between biopsies, type of surgery, and pathological tumor parameters was analyzed. Recurrence-free survival (RFS) and overall survival (OS) were analyzed using the Kaplan–Meier method. CNB had a sensitivity of 77.95% and a specificity of 80.97% for identifying luminal B tumors in CNB, compared with the final molecular subtype determination after surgery. The correlation of Ki67-LI between CNB and SR was moderate (ROC-AUC 0.8333). Specificity and sensitivity for CNB to correctly define molecular subtype of tumors according to SR were significantly associated with tumor grade, immunohistochemical progesterone receptor (PR) and p53 expression (*p* < 0.05). Agreement of molecular subtype did not significantly impact RFS and OS (*p* = 0.22 for both). The identified factors likely mirror intratumoral heterogeneity that might compromise obtaining a representative CNB. Our results challenge the robustness of a single CNB-driven measurement of Ki67-LI to identify luminal B breast cancer of low (G1) or intermediate (G2) grade.

## Introduction

Reliable determination of molecular subtype is indispensable for prognostication and treatment decision in breast cancer (BC) [1, 2]. Especially in estrogen receptor (ER)–positive and human epidermal growth factor 2 (HER2)–negative luminal BC, assessment of molecular subtype is of critical prognostic importance [3–9]. Current St. Gallen guidelines confirm the role of the proliferation marker Ki67 labeling index (Ki67-LI) in discriminating good prognosis “luminal A” (LumA) from “luminal B” (LumB) disease with less favorable prognosis [2]. In the absence of molecular assays, Ki67-LI still remains a main factor governing molecular subtype determination and (neo-)adjuvant treatment choice in early luminal-type BC [1, 10–14]. However, reproducible and clinically valid Ki67-LI determination in core needle biopsy (CNB) might be biased by technical difficulties, assessment methods, or intratumoral heterogeneity. Exact measures of standardization such as a cutoff value or compensating for intratumoral heterogeneity remain elusive. Although digital image analysis seems a promising tool to facilitate robust and reliable determination of Ki67-LI, additional challenges still remain to be addressed [15, 16]. At present, no universal Ki67-LI cutoff levels to define molecular subtype in luminal BC are specified, and discrepancies regarding implications on clinical decisions persist [1, 17–19]. Whereas cutoff values can be statistically validated, the influence of intratumoral heterogeneity on the reliability of Ki67-LI in CNB remains to be resolved.

Recommendations to standardize Ki67-LI assessment recognized the need for further studies to evaluate comparability of Ki67-LI between CNB and whole tumor slides of surgical resection (SR) specimens [17]. We therefore evaluated the reliability of Ki67-LI to define molecular subtype by analyzing the concordance between CNB and SR specimens in 484 early untreated luminal-type BC patients. We investigated clinicopathological factors and possible sampling-associated confounders, such as CNB method and time between CNB and surgery that might impact the reliability of molecular subtype/Ki67-LI determination in CNB. Our results might help to improve the interpretation of CNB-derived measurements as prognostic indicators and outcome predictors in luminal BC. The identification of factors that predict discrepancies between CNB and SR could aid the targeted implementation of molecular assays in luminal BC cases where reliable molecular subtype determination and neoadjuvant therapy indication require additional analyses.

## Patients and methods

### Patients

Patients (*n* = 484) with ER+/HER2− invasive BC at the Medical University of Vienna (MUW) were included in this retrospective analysis. Study procedures were approved by the Institutional Review Board of the MUW (1245/2017). Patients with primary operable luminal BC stages I–III who received diagnostic CNB and curative surgery without neoadjuvant therapy between 2010 and 2012 were eligible to participate. Age, menopausal status, type of surgery (breast conserving vs. mastectomy), and previous BC history were recorded for each patient. Clinical and pathological patient characteristics are described in Table 1. Follow-up data was available for 390 (80.6%) patients. At a median follow-up of 62.6 months (ranged 1–105 months), 63 (16.2%) patients had relapsed and 63 patients (16.2%) had died.

**Table 1:** Patients’ characteristics: data are presented as median (minimum-maximum) or as absolute frequencies (percentages)

Clinicopathological parameters	*N* (%)
Age (years)	
Median (min-max)	62.5 (29.8–92.7)
Menopausal status	
Premenopausal/postmenopausal/unknown	93 (19.2%)/379 (78.3%)/12 (2.5%)
Breast cancer history	
No previous BC/recurrent BC	432 (89.3%)/52 (10.8%)
CNB method	
US-guided/stereotactic/MR-guided/palpatory	389 (80.4%)/21 (4.3%)/50 (10.3%)/24 (5.0%)
BI-RADS assessment category	
IV/V/unknown	160 (33.1%)/258 (58.9%)/39 (8.1%)
Surgery time interval (STI, days)	
Median (min-max)	25.0 (2–105)
Type of surgery	
Breast conserving surgery/mastectomy	362 (74.8%)/122 (25.2%)
Tumor type	
NST/lobular/mixed/other	394 (81.4%)/70 (14.5%)/4 (0.8%)/16 (3.3%)
Grade	
G1/G2/G3/GX	CNB: 142 (29.3%)/247 (51.1%)/94 (19.4%)/1 (0.2%) SR: 117 (24.2%)/253 (52.3%)/114 (23.6%)/---
Molecular subtype	
LumA/LumB	CNB: 207 (42.8%)/277 (57.2%) SR: 195 (40.3%)/289 (59.7%)
In situ (DCIS) component in CNB	
Yes/no	212 (43.8%)/272 (56.2%)
Tumor size	
pT1/pT2/pT3/pT4	338 (69.8%)/128 (26.4%)/14 (2.9%)/4 (0.8%)
Focality	
Unifocal tumor/multifocal tumor/unknown	364 (75.2%)/119 (24.6%)/1(0.2%)
Lymph node status	
pN0/pN1a/pN2a/pN3a/pNx	325 (67.1%)/100 (20.7%)/25 (5.2%)/18 (3.7%)/16 (3.3%)
Lymphovascular invasion	
Absent/mild/severe	CNB: 465 (96.1%)/19 (3.9%)/--- SR: 340 (70.2%)/138 (28.5%)/6 (1.3%)

### Radiology

All patients underwent pre-operative either stereotactic vacuum-assisted (9G), ultrasound core needle (14G), or magnetic resonance imaging (MRI) vacuum-assisted (9G) guided breast biopsy according to the European Society of Breast Imaging (EUSOBI) guidelines [20]. In some patients, CNB was performed under no image guidance (palpation). Documented parameters included Breast Imaging Reporting and Data System (BI-RADS) assessment category of image-detected lesions, CNB method, and surgery time interval (STI) between CNB and surgery (Table 1).

### Pathology and immunohistochemistry

Workup was carried out according to the EU guidelines [21, 22] and the WHO classification [23]. Histopathological tumor grade (G) [24, 25], tumor size (pT), multifocality, lymph node (pN) status, and presence of peritumoral lymphovascular invasion (LVI) were determined by two experienced pathologists (ML, ZBH). All cases were reviewed together; discrepant cases were discussed to reach consensus. All grading parameters (tubuloglandular differentiation, nuclear pleomorphy, and mitotic count) were recorded separately for CNB and SR, whereas measurements in SR were defined as gold standard.

Assessment of ER, progesterone receptor (PR), HER2, Ki67-LI, and p53 was performed using Ventana BenchMark Ultra (Ventana, Tucson, AR, USA) according to the ASCO/USCAP guidelines [26, 27]. Ki67-LI was determined as described previously [28]. The entire tumor area was evaluated by estimation, yielding an average Ki67-LI score. LumA molecular subtype was defined by a Ki67-LI of < 20%. To enable comparability, % scores of ER, PR, Ki67, and p53 were normalized to ten-percentile values. Accordingly, cases with Ki67-LI values between 10 and 14% were regarded as LumA disease, whereas the definition of luminal B breast cancer included cases with values between 15 and 20%.

### Statistical analyses

Continuous data are presented as median, minimum, and maximum due to skew distributions. Categorical data are presented as absolute frequencies and percentages. Sensitivities to correctly predict LumB molecular subtype in SR and specificities to predict LumA molecular subtype by CNB are given partly together with 95% confidence intervals according to the method of Wilson. To assess diagnostic ability of CNB to predict SR over several CNB cut-points, a receiver operating characteristic (ROC) curve and its area under the curve are given.

Associations between two binary variables are tested by chi-square test or Fisher’s exact test in case of small cell numbers. To test ordinal variables between LumA and LumB molecular subtype, a trend chi-square test was used and an exact version was used in case of small numbers.

Recurrence-free survival was defined as the interval between the CNB and the first evidence of relapse at any site or incidence or contralateral breast cancer. Overall survival was defined as the interval between CNB and death. Survival rates were estimated with the use of the Kaplan–Meier method.

All *p* values are two-sided and *p* ≤ 0.05 was considered significant. Calculations were performed by the statistical software SAS© (version 9.4, SAS Institute Inc., Cary, NC, USA).

## Results

### Concordance of Ki67-LI and molecular subtype between CNB and SR

**Fig. 1:**
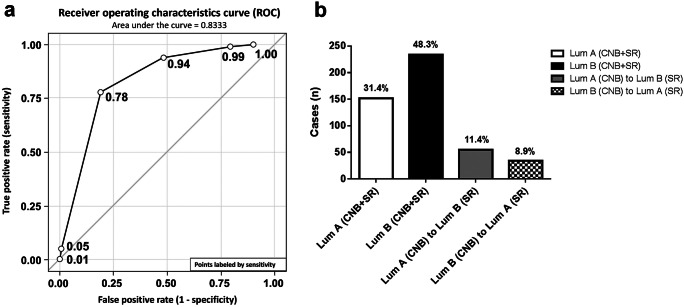
a Receiver operating characteristics (ROC) curve for luminal IST by CNB. Numbers given are true positive rate and correspond to CNB cutoff values of < 5, < 10, < 20, < 30, < 40, and < 50, respectively. b Agreement of luminal IST determination in core needle biopsy (CNB) and surgical resection (SR) specimens. Using a cutoff value of < 20% for LumA IST, 386 cases (79.8%) were correctly classified in CNB; 98 cases (20.5%) showed discordant IST

Median Ki67-LI was 21.4% and 21.7% for CNB and SR, respectively. A substantial agreement of Ki67-LI between CNB and SR specimens was observed. The ROC for molecular subtype in CNB to correctly diagnose LumA in SR (Ki67-LI < 20%) resulted in an area under the curve (AUC) of 0.8333 (Fig. 1a), indicating moderate association. When applying a cutoff point of CNB Ki67-LI < 20% for LumA molecular subtype, 152 of 195 LumA cases were diagnosed correctly (sensitivity 77.95%; 95% CI 71.62–83.20%) and 43 tumors were falsely classified as LumB by CNB. Two hundred thirty-four out of 289 LumB cases were correctly classified as Ki67-LI ≥ 20% (specificity 80.97%; 95% CI 76.05–85.08%), and 55 cases were falsely classified as LumA by CNB (Fig. 1b).

**Fig. 2:**
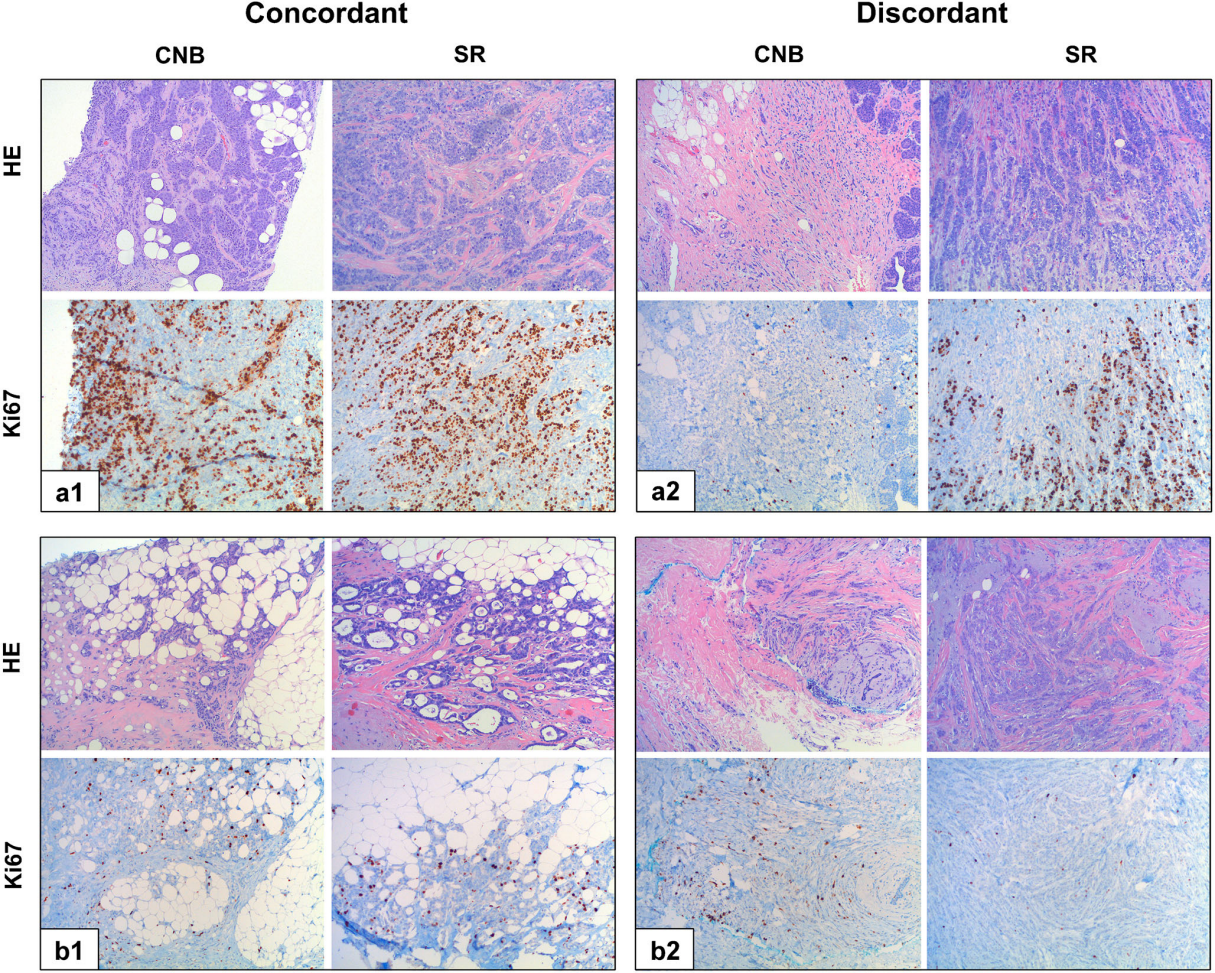
Hematoxylin and eosin (HE) and Ki67-LI immunohistochemical stainings showing breast cancer cases displaying concordant and discordant luminal IST and Ki67-LI in CNB and SR, respectively (× 100 magnification). a HE and respective Ki67-LI IHC slides of two patients with concordant Ki67-LI when comparing CNB and SR. A1 Ki67-LI 60% indicating LumB IST in both CNB and SR; A2 10% Ki67 indicating LumA IST in both CNB and SR. b HE and respective Ki67 IHC slides of two patients with discordant luminal IST and Ki67-LI when comparing CNB and SR. B1 Ki67 10% (LumA) in CNB, 30% (LumB) in SR; B2 20% (LumB) Ki67-LI in CNB, 5% (LumA) in SR.

Agreement of molecular subtype determination by Ki67-LI was observed in 386 (79.8%) patients. In 98 (20.3%) patients, Ki67-LI in CNB and SR were discordant. Examples are shown in Fig. 2.

### CNB-related factors influencing agreement

In our analysis, we identified tumor grade (including all separate grading components), lower PR expression, higher p53 expression, and LVI as tumor-related factors in CNB that significantly influenced agreement of molecular subtype. A dependence between ER expression in CNB and Ki67-LI for LumA and LumB tumors could not be demonstrated, mainly due to small group sizes with ER < 80% in CNB (7 LumA tumors and 14 LumB tumors). Results are shown in detail in Table 2.

**Table 2:** Tumor-related factors in CNB influencing agreement of luminal IST

		Intrinsic subtype (*n*)		Specificity (%) *	*p* value	Intrinsic subtype (*n*)		Sensitivity (%) **	*p* value
		LumA^SR^				LumB^SR^			
		LumA^CNB^	LumB^CNB^			LumA^CNB^	LumB^CNB^		
	Total	152	43	78.0		55	234	81.0	
CNB-grade	G1	85	12	87.6	0.0007	28	17	37.8	< 0.0001
	G2	67	30	69.1		24	126	84	
	G3	0	1	0		3	90	96.8	
	Missing						1		
SR-grade	G1	78	11	-	0.0061	16	12	-	< 0.0001
	G2	73	32			35	113		
	G3	1	0			4	109		
CNB-nuclear pleomorphy	1	39	3	92.9	0.0066	11	6	35.3	< 0.0001
	2	108	37	74.5		43	164	79.2	
	3	5	3	62.5		1	64	98.5	
CNB-mitotic count	1	143	29	83.1	0.0003	49	87	64.0	< 0.0001
	2	8	11	42.1		4	56	93.3	
	3	1	1	50.0		1	87	98.9	
	Missing	0	2	0		1	4	80.0	
CNB lymphovasc. invasion	No	152	43	78.0	-	54	216	80.00	0.1390
	Yes	0	0	-		1	18	94.74	
CNB ER	10–30%	0	1	0	0.1027	0	1	100	0.3548
	40–70%	4	2	66.7		1	12	92.3	
	80–100%	148	40	78.7		54	221	80.3	
CNB PR	neg.	22	7	75.9	0.7984	2	30	93.8	0.0006
	10–30%	14	1	93.3		2	39	95.1	
	40–70%	37	12	75.5		16	65	80.2	
	80–100%	79	23	77.5		35	100	74.1	
CNB p53	neg.	148	40	78.72	0.0868	51	172	77.13	0.0017
	10–30%	4	2	66.67		4	37	90.24	
	40–60%	0	0	-		0	16	100	
	70–100%	0	1	0		0	9	100	

^*^Specificity is the percentage of correctly diagnosed LumA patients by CNB

^**^Sensitivity is the percentage of correctly diagnosed LumB patients by CNB

Missing values were ignored in statistical tests

### SR-related factors influencing agreement

In SR specimens, tumor grade (including all separate grading components), tumor size (pT), multifocality, lymph node involvement (pN), LVI, and performed mastectomy were identified as tumor-related factors that significantly influenced agreement of molecular subtype. Results are shown in detail in Table 3.

**Table 3:** Tumor-related factors in SR influencing agreement of luminal IST

		Intrinsic subtype (n)		*p* value	Intrinsic subtype (n)		*p* value
		LumA^SR^			LumB^SR^		
		LumA^CNB^	LumB^CNB^		LumA^CNB^	LumB^CNB^	
	Total	152	43		55	234	
SR-grade	G1	78	11	0.0061	16	12	< 0.0001
	G2	73	32		35	113	
	G3	1	0		4	109	
SR-glandular differentiation	1	39	7	0.2024	5	7	0.0009
	2	43	12		21	53	
	3	70	24		29	173	
	Missing	0	0	-	0	1	-
SR-nuclear pleomorphy	1	34	4	0.0137	5	4	< 0.0001
	2	103	30		36	102	
	3	15	9		14	127	
	Missing	0	0	-	0	1	-
SR-mitotic count	1	142	39	0.7627	36	91	< 0.0001
	2	9	4		14	53	
	3	1	0		5	89	
	Missing	0	0	-	0	1	-
SR pT	pT1	124	36	0.7466	37	141	0.3357
	≥ pT2	28	7		18	93	
SR lymphovascular invasion	no	126	40	0.0993	38	136	0.0945
	Yes—mild	26	3		17	92	
	Yes—severe	0	0	-	0	6	-
Multifocality	No	124	29	0.0465	45	166	0.1110
	Yes	28	14		10	67	
	Missing				0	1	-
SR-pN	Negative	114	36	0.4132	34	141	0.7858
	pN1a	29	6		13	52	
	pN2a	2	0		4	19	
	pN3a	4	1		2	11	
	Missing	3	0	-	2	11	-
Breast conserving surgery	Mastectomy	31	17	0.0101	9	65	0.0809
	BCS	121	26		46	169	

Missing values were ignored in statistical tests

### Prognostic significance of molecular subtype agreement

**Fig. 3:**
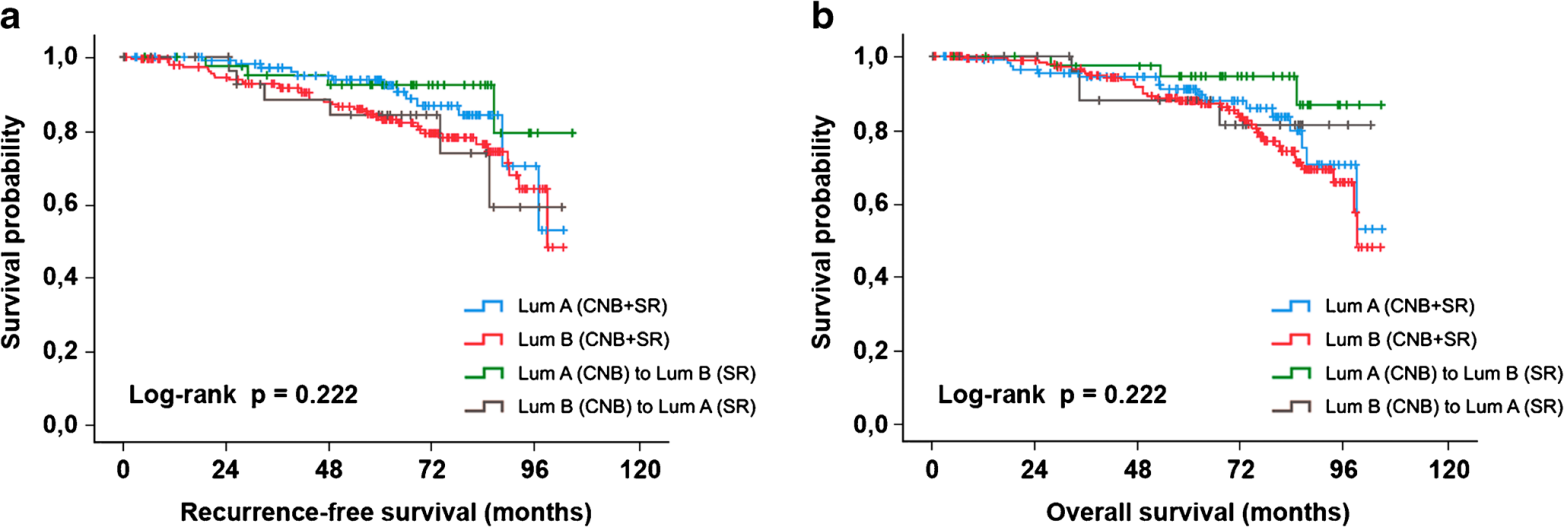
Recurrence-free (a) and overall (b) survival of patients according to IST in CNB/SR. Correctly diagnosed cases included 177 LumA tumors (CNB + SR) and 199 LumB tumors (CNB + SR). Within the discordant cases, 43 LumA tumors in CNB were upgraded to LumB by SR, and in 31 LumB, tumors in CNB were downgraded to LumA by SR.

Survival data was available for 390 patients, of whom 177 (30%) were correctly diagnosed with luminal A tumors and 199 (51%) were correctly diagnosed with luminal B disease in CNB. In 43 patients (11.2%), a LumA tumor in CNB was upgraded to LumB by SR and in 31 patients (7.9%), a LumB tumor in CNB was downgraded to LumA by SR. Kaplan–Meier analyses revealed no significant differences regarding RFS and OS (log-rank test, *p* = 0.22, respectively). However, patients with correctly diagnosed LumB tumors tended to have a worse RFS and OS, as shown in Fig. 3.

## Discussion

The present study analyzed concordance of Ki67-LI between corresponding CNB and SR in BC with respect to defining molecular subtype. Our aim was to identify method- and tumor-related factors that influence reliability of Ki67-LI/molecular subtype determination in CNB. Although smaller studies have previously been conducted [29–31], our study is distinguished by the inclusion of patients with luminal BC exclusively, as Ki67-LI has a therapeutic consequence only in these patients [30]. Of particular importance is the reliable identification of LumB disease in CNB, as in these patients, neoadjuvant chemotherapy is often indicated.

In our study, 79.8% of tumors were reliably classified according to molecular subtype, indicating a moderate agreement. Discrepancies in Ki67-LI between CNB and SR samples have been reported, even when no neoadjuvant systemic therapy was applied [32]. Sampling errors as well as intratumoral heterogeneity leading to a non-representative CNB have been discussed as potential confounding factors [17]. The abundance of tumor cells in SR compared with CNB can represent intratumoral heterogeneity to a higher degree. Therefore, the representativity of CNB might be questionable [29, 33, 34]. Ki67-LI in SR was more accurately mirrored by CNB than by tissue microarrays, without direct influence of CNB size on reliability [29, 33–35]. Recent studies addressing intratumoral heterogeneity demonstrated that inhomogeneous distribution of tumor proliferation within single tumors exceeded Ki67-LI variation between individual luminal-type tumors [36].

Further factors such as post-acquisition handling have been considered to be of influence as well. A prolonged time span between extraction and fixation leading to hypoxic tissue damage and in succession to apoptosis of tumor cells and degradation of the Ki67 nuclear protein might result in a lower Ki67-LI in SR samples [29, 37].

We demonstrated that the CNB method did not significantly influence reliability of molecular subtype determination, whereas MRI-guided biopsies showed the lowest concordance rates (data not shown). By evaluating the influence of the time interval between CNB and surgery, we analyzed whether wound healing processes after CNB impact local tumor proliferation. Previous studies suggested that surgery time interval might influence Ki67-LI change after CNB, longer STI leading to a higher increase in Ki67-LI [38]. In our analysis, surgery time interval did not interfere with Ki67-LI and molecular subtype concordance and reliability. However, median surgery time interval differed significantly in the two studies (4.5 days reported by Chen at al. vs. 25 days in this study, respectively), which provides a possible explanation for this discrepancy: a transient rise in Ki67-LI caused by wound healing processes is likely abated after a longer STI.

In our study, further clinicopathological factors, most importantly G and size, PR and p53 expression influenced sensitivity and specificity of molecular subtype determination. These factors are likely indicators of intratumoral heterogeneity and might therefore predict reliability of molecular subtype assessment in CNB. Previous analyses confirmed that adverse clinicopathological factors such as high G, LVI, and high p53 expression were significantly associated with a high Shannon index regarding copy number variation of oncogenes, implicating these factors as indicators of intratumoral heterogeneity [39].

ER-negative tumors display higher concordance rates between CNB and SR than ER-positive tumors [30]. In our study, PR negativity was significantly associated with increased concordance in luminal BC. Furthermore, inferior agreement was found in G1 and G2 tumors compared with G3 tumors. We also reported a lower Ki67-LI concordance rate and lower luminal molecular subtype agreement in comparison with the distinction between luminal and non-luminal subtype [30].

We now report that additional factors, such as multifocality, PR, and p53 expression, also affect sensitivity of Ki67-LI assessment. Considering these factors is likely to increase the acuity of molecular subtype determination.

In our study, all separate grading components, namely glandular differentiation, nuclear pleomorphy, and mitotic count, inflicted a significant impact on correctly classifying luminal molecular subtype. Although applying histological grade and mitotic index might reliably identify LumA tumors, Ki67-LI is needed to correctly classify LumB tumors [40]. In our study, specificity to identify LumA tumors decreased with increasing histological grade. These findings are in line with previous studies reporting frequent underestimation of tumor grade in CNB, whereas reliable grading showed significant association with CNB size [36].

At present, no uniform cutoff levels for Ki67-LI have been defined and discrepancies regarding thresholds and their implications for clinical decisions remain [1, 2, 17–19, 41]. Regarding this problem, an expert panel was not able to stipulate an ideal cutoff point for routine use [17]. Association of Ki67-LI values between 10 and 20% with BC outcome has been proven in a meta-analysis [42]. Inter-observer variability was most pronounced between 10 and 30% positivity, where clinically valid cutoff values are suggested [43]. The normalization to 5-percentile values and a variable cutoff may solve this problem, depending on the clinical purpose [32]. For the present study, we chose a cutoff of ≥ 20 for LumB BC, as previously validated in a clinical trial [28].

Our study revealed no statistically significant differences in RFS and OS depending on agreement of molecular subtype between CNB and SR, which might be explained by the limited cohort size and the low number of events.

Previous studies suggested that digital image analysis vs. visual determination might improve molecular subtype determination by immunohistochemistry [44]; however, comparative studies using digital image analysis in assessing Ki67-LI in CNB and SR are scarce. In these smaller cohorts, the reported agreement between CNB and SR was comparable with our results, although digital image analysis slightly improved determination [45, 46]. Notably, none of these studies investigated the impact of molecular subtype/Ki67-LI determination on patient survival.

In our study, pathological factors such as tumor grade, size, PR, and p53 expression significantly influenced agreement of Ki67-LI and luminal molecular subtype between CNB and SR. These factors mirror tumor heterogeneity and can objectivize molecular subtype determination in CNB.

More importantly, our results question the robustness of a single CNB-driven measurement of Ki67 in luminal BC of low (G1) or intermediate (G2) histological grade and warrant further investigations to improve the validity of molecular subtype determination in these cases.

## Data Availability

The datasets generated during and/or analyzed during the current study are not publicly available due to legal personal data protection issues but are available from the corresponding author on reasonable request.
